# Lesão pulmonar autoinflingida: é possível identificar o risco? Relato de caso

**DOI:** 10.5935/0103-507X.20210061

**Published:** 2021

**Authors:** Joaquín Pérez, Javier Hernán Dorado, Emiliano Navarro, Matías Accoce

**Affiliations:** 1 Sanatorio Anchorena de San Martín - Buenos Aires, Argentina.; 2 Hospital General de Agudos “Carlos G. Durand” - Buenos Aires, Argentina.; 3 Centro del Parque - Buenos Aires, Argentina.; 4 Hospital de Quemados “Dr. Arturo Umberto Illia”- Buenos Aires, Argentina.; 5 Universidad Abierta Interamericana - Buenos Aires, Argentina.

**Keywords:** Lesão pulmonar induzida por ventilador, Suporte ventilatório interativo, Respiração artificial, Síndrome do desconforto respiratório, Monitorização

## Abstract

A respiração espontânea pode ser prejudicial para pacientes com pulmões previamente lesados, especialmente na vigência de síndrome do desconforto respiratório agudo. Mais ainda, a incapacidade de assumir a respiração totalmente espontânea durante a ventilação mecânica e a necessidade de voltar à ventilação mecânica controlada se associam com mortalidade mais alta. Existe uma lacuna no conhecimento em relação aos parâmetros que poderiam ser úteis para predizer o risco de lesão pulmonar autoinflingida pelo paciente e detecção da incapacidade de assumir a respiração espontânea. Relata-se o caso de um paciente com lesão pulmonar autoinflingida e as correspondentes variáveis, básicas e avançadas, de monitoramento da mecânica do sistema respiratório, além dos resultados fisiológicos e clínicos relacionados à respiração espontânea durante ventilação mecânica. O paciente era um homem caucasiano com 33 anos de idade e história clínica de AIDS, que apresentou síndrome do desconforto respiratório agudo e necessitou ser submetido à ventilação mecânica invasiva após falha do suporte ventilatório não invasivo. Durante os períodos de ventilação controlada, adotou-se estratégia de ventilação protetora, e o paciente mostrou evidente melhora, tanto do ponto de vista clínico quanto radiográfico. Contudo, durante cada período de respiração espontânea sob ventilação com pressão de suporte, apesar dos parâmetros iniciais adequados, das regulagens rigorosamente estabelecidas e do estrito monitoramento, o paciente desenvolveu hipoxemia progressiva e piora da mecânica do sistema respiratório, com deterioração radiográfica claramente correlacionada (lesão pulmonar autoinflingida pelo paciente). Após falha de três tentativas de respiração espontânea, o paciente faleceu por hipoxemia refratária no 29° dia. Neste caso, as variáveis básicas e avançadas convencionais não foram suficientes para identificar a aptidão para respirar espontaneamente ou predizer o risco de desenvolver lesão pulmonar autoinflingida pelo paciente durante a ventilação de suporte parcial.

## INTRODUCTION

Spontaneous breathing (SB) can be potentially deleterious in patients with previously injured lungs. Specifically, in moderate-severe acute respiratory distress syndrome (ARDS), vigorous inspiratory effort may amplify the stress-strain applied to the dependent lung regions and produce local inflammatory mediators release with systemic consequences, the so-called self-inflicted lung injury (P-SILI).^(1)^ Despite the potential relevance of P-SILI, it has only been demonstrated in animal models and controlled laboratory research studies with scarce descriptions in clinical practice.^(2, 3)^

The prolongation of controlled mechanical ventilation (MV) time increases the risk of respiratory infections and diaphragmatic weakness, which may hamper ventilator weaning. On the other hand, the premature adoption of partial ventilatory support may be associated with a high respiratory drive and cause respiratory failure with the consequent need to switch back to controlled MV, which has been associated with higher mortality and worse outcomes in ARDS.^(4, 5)^

There is a gap of knowledge regarding which ventilatory variables allow clinicians to detect the aptitude to breathe spontaneously and to identify the risk of P-SILI in patients recovering from ARDS.^(4, 6)^ Single parameters, such as oxygenation, respiratory drive, respiratory system (RS) mechanics and the work of breathing (WOB), have been proposed as potential promoters of P-SILI;^(2, 4, 7)^ however, all of them remain controversial and there is no strong scientific evidence in their favor.^(4)^

We report conventional basic and advanced monitoring variables of RS mechanics in a patient who developed P-SILI during the partial ventilatory support phase with the corresponding physiological and clinical outcomes related to SB.

## CASE REPORT

The patient was a 33-year-old Caucasian man with a medical history of AIDS and 1 year without treatment who attended the emergency room with a three-weeks progressive dyspnea followed by treatment with levofloxacin for 5 days and amoxicillin/clavulanic acid for 7 days with no adequate response.

**Figure 1:**
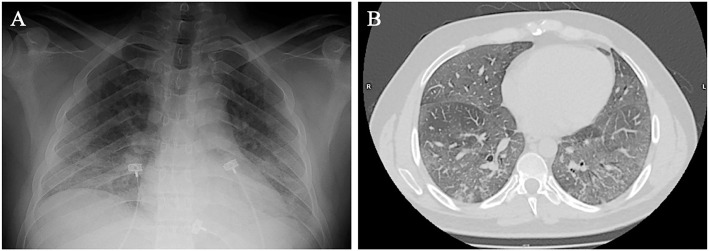
Chest radiography (A) and computed tomography (B) at admission.

At admission, he presented with tachypnea, fever 39.1°C, dry cough and hypoxemia. Chest radiography and computed tomography showed bilateral interstitial pulmonary infiltrates with no localized alveolar opacities (Figure 1). Immediately, noninvasive ventilation (NIV) was implemented; sputum, blood and urine cultures were taken and empiric antibiotics were initiated.

He was admitted to the intensive care unit (ICU) using NIV, with a Glasgow coma scale score of 15/15, respiratory rate 28-34 breaths per minute (bpm), dyspnea score 8/10 (zero if no dyspnea at all; ten if greater imaginable dyspnea), comfort 8/10, use of accessory muscles and a *Heart Rate, Acidosis, Consciousness, Oxygenation, and Respiratory Rate* (HACOR) score of 4 points.^(8)^ After 1 hour of NIV, arterial blood gases showed pH 7.38, partial pressure of carbon dioxide (PaCO_2_) 38mmHg, partial pressure of oxygen (PaO_2_) 78.1mmHg, bicarbonate (HCO_3_) 22.2mEq/L, Base Excess (BE)-2.4mEq/L and a ratio of PaO_2_ to the fraction of inspired oxygen (PaO_2_/FiO_2_) 156.2mmHg.

Considering the clinical presentation features, we decided to switch from NIV to high-flow nasal cannula (HFNC) using a 60L/minute flow rate and FiO_2_ 0.50. Initially, the patient reduced the respiratory rate to 23bpm, and the oxygenation, dyspnea and comfort improved. The ROX index after 1 hour of HFNC was 8.33.^(9)^

After 48 hours of HFNC, the patient increased the WOB, and the oxygenation worsened, so it was necessary to proceed to endotracheal intubation and invasive MV.

### First period of controlled mechanical ventilation

During invasive MV, we carried out advanced monitoring of the RS mechanics through esophageal manometry. Initially, we implemented protective MV using a tidal volume (VT) of 4 - 6mL/kg of predicted body weight (PBW), positive end expiratory pressure (PEEP) titration according to the best RS compliance (C_rs_), a target of plateau pressure (Pplat) < 30cmH_2_O, driving airway pressure (∆P_aw_) < 15cmH_2_O and driving transpulmonary pressure (∆P_L_) < 12cmH_2_O, deep sedation, neuromuscular blocking agents (NMBAs) and prone positioning (PP) (Figure 1S - Supplementary material).

On day 4 of invasive MV, after a clear improvement in oxygenation, the sedation levels were reduced in an attempt to begin the partial ventilatory support phase and he was switched from controlled MV to pressure support ventilation (PSV).

### First period of partial ventilatory support

**Figure 2:**
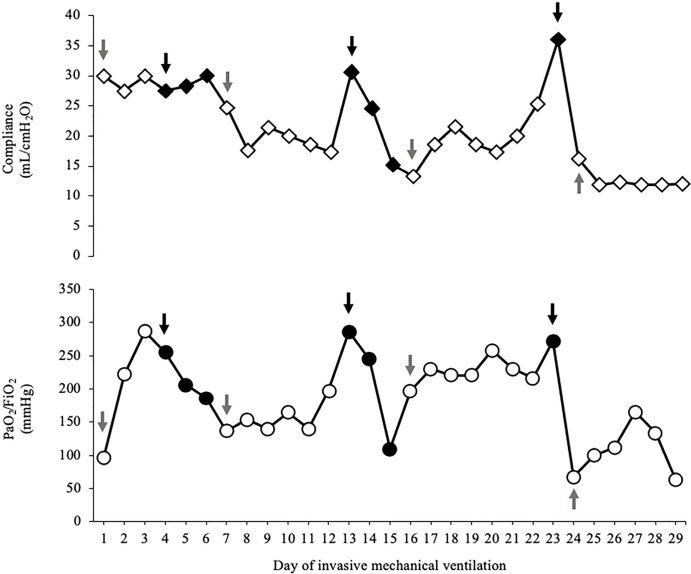
Evolution of respiratory system compliance (diamonds) and oxygenation (circles) during invasive mechanical ventilation. Compliance in volume-controlled continuous mandatory ventilation and pressure support ventilation was evaluated in static conditions through an end-inspiratory occlusion of 2 seconds, discarding respiratory muscle activation during the procedure, particularly during pressure support ventilation. Gray arrows show the day when controlled mechanical ventilation started, white empty symbols represent days of controlled mechanical ventilation in volume controlled - continuous mandatory ventilation, black arrows show the day where spontaneous breathing with pressure support ventilation started, and black full symbols represent days of spontaneous breathing with pressure support ventilation. Even though spontaneous breathing was maintained for only a few hours on days 15 and 23, we decided to express the beginning of the controlled ventilation phase in the graph on days 16 and 24, respectively, because they represent full passive ventilation days (see main text). Notably, the oxygenation worsened during every spontaneous breathing period. Compliance of the respiratory system followed the same behavior except in the first partial support cycle. PaO_2_ - arterial partial pressure of the oxygen; FiO_2_ - fraction of inspired oxygen.

**Figure 3:**
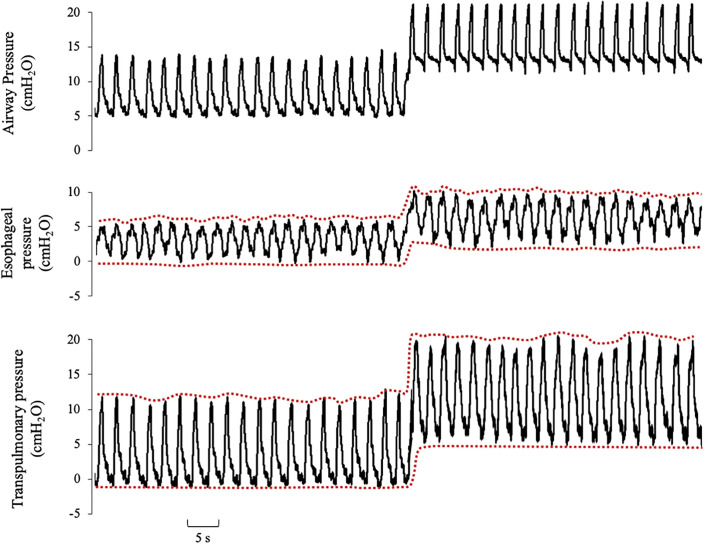
Esophageal and transpulmonary pressure response to a positive end expiratory pressure step of 10cmH_2_O in pressure support ventilation.

Figure 2 shows the evolution of oxygenation and C_rs_ during this period. In addition, table 1 describes the daily ventilatory settings and monitoring during PSV. We carried out a decremental PEEP titration trial to optimize our ventilatory strategy. We observed that higher PEEP levels did not ameliorate the esophageal pressure swing (∆P_es_) or the ∆P_L_ and they even seemed to increase (Figure 3); therefore, we prioritized lower PEEP values to reduce the stress and mechanical energy applied to the lungs.

**Table 1:** Basic and advanced monitoring variables during spontaneous breathing days on pressure support ventilation

	Day 4	Day 5	Day 6	Day 13	Day 14	Day 15	Day 23
Support level (cmH_2_O)	8	6	6	10	12	10	6
PEEP (cmH_2_O)	6	5	5	5	5	5	5
FiO_2_	0.35	0.35	0.45	0.35	0.50	0.65	0.5
Tidal volume (mL/kg PBW)	6.1 ± 0.5	5 ± 0.3	5 ± 0.4	6.1 ± 0.2	5.6 ± 0.3	5.8 ± 0.5	6.7 ± 0.3
Respiratory rate	25	24	27	25	30	48	22
P0.1 (cmH_2_O)	1.44 ± 0.4	1.8 ± 0.2	0.86 ± 0.1	1.5 ± 0.2	1.7 ± 0.3	3.5 ± 0.7	3 ± 0.8
Muscular pressure index (cmH_2_O)	3	6	5	2	3	13	6
Dynamic Δ P_tp_(cmH_2_O)	13.7 ± 0.8	11.6 ± 1.3	11.0 ± 1.3	14.3 ± 1.8	16.4 ± 1.9	22.2 ± 1.6	14.5 ± 1
Δ P_es_(cmH_2_O)	4.9 ± 0.9	5.1 ± 1.4	5.4 ± 0.9	4.0 ± 1.1	4.3 ± 0.8	13.0 ± 1.5	7.3 ± 1.8
PTP_es_(cmH_2_O.seg/minute)	98.2 ± 17.8	105 ± 15.3	116.1 ± 22.7	80,4 ± 20.7	103.6 ± 21.3	350.5 ± 42.4	103 ± 7.7

Esophageal pressure monitoring was carried out three times a day for 30 - 45 minutes. Data analyses were performed after the identification of a sequence of breaths deemed representative during the standard settings of each day. The temporal sequence is expressed as the day number of invasive mechanical ventilation where spontaneous breathing was present.

PEEP - positive end expiratory pressure; FiO_2_ - fraction of inspired oxygen; PBW - predicted body weight; P0.1 - pressure during the first 100 milliseconds of inspiratory occlusion; Ptp - transpulmonary pressure; Pes - esophageal pressure;

PTPes - esophageal pressure-time product. Values are expressed as absolute values or mean ± standard deviation.

On day 6 of invasive MV, the patient met the classic weaning criteria; thus, we carried out a spontaneous breathing trial (SBT) with 5cmH_2_O of pressure support level to assess the chance for extubation. The patient failed the SBT after 20 minutes due to hypoxemia.

On day 7 of invasive MV, due to persistent worsening of oxygenation, we carried out a computed tomography, which showed clear progression of the lung injury with the appearance of alveolar bilateral diffuse infiltrate primarily in the dependent lung regions (Figure 2S - Supplementary material). In this context, we decided to reinstitute controlled MV and deep sedation levels.

### Second period of controlled mechanical ventilation

In this period, we electively adopted PP cycles from 16 to 20 hours a day with the aim of achieving oxygenation stability, minimizing the risk of lung injury and projecting a new SB period under more favorable conditions (Figure 3S - Supplementary material).^(10, 11)^ The supine position was maintained for 4 - 5 hours between PP cycles.

On day 8 of invasive MV, a bronchoalveolar lavage showed positive results for cytomegalovirus and *Acinetobacter baumannii*. Thus, directed antibiotics were immediately initiated. It is important to highlight that during this period, it was necessary to reduce the VT to 4mL/kg PBW to maintain protective ventilation parameters (Figure 1S - Supplementary material).

On day 13 of invasive MV, after a clear improvement of oxygenation (PaO_2_/FiO_2_ = 286mmHg) and C_rs_ (30mL/cmH_2_O), we attempted a new sedation vacation (Figure 2).

### Second period of partial ventilatory support

**Figure 4:**
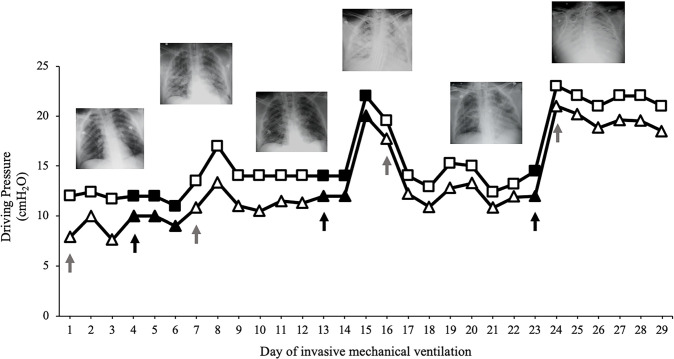
Evolution of the airway driving pressure (squares) and transpulmonary driving pressure (triangles) during the invasive mechanical ventilation days. Airway and transpulmonary driving pressure in volume controlled continuous mandatory ventilation and pressure support ventilation were evaluated in static conditions through an end-inspiratory occlusion of 2 seconds, discarding respiratory muscle activation during the procedure, particularly in pressure support ventilation. Gray arrows show the day where controlled mechanical ventilation started, white empty symbols represent days of controlled mechanical ventilation in volume controlled continuous mandatory ventilation, black arrows show the day where spontaneous breathing in pressure support ventilation started, and black full symbols represent days of spontaneous breathing in pressure support ventilation. Even though spontaneous breathing was maintained for only for a few hours on days 15 and 23, we decided to express the beginning of controlled ventilation in the graph on days 16 and 24 because it represents a full passive ventilation day (see main text). Of note, a clear radiographic correlation was observed between the days of spontaneous breathing on pressure support ventilation and the appearance of new bilateral diffuse infiltrates after the end of each partial support period. During controlled ventilation cycles, the radiographic pattern improved clearly.

In the first 48 hours of partial support, the patient maintained safe spontaneous effort values in PSV (Table 1, Figure 4). However, on the morning of the third day of SB, the patient showed a sudden remarkable increase in respiratory drive and WOB, leading to higher ∆P_L_ (Table 1).

Initially, this change in the clinical condition was attributed to fever, asynchronies, anxiety and pain, so we treated them using antipyretic, anxiolytic and analgesic medication with a poor response. In this context, we observed clear oxygenation, RS mechanics and radiographic deterioration (Table 1, Figures 2 and 4), so it was necessary to re-initiate controlled MV and deep sedation.

### Third period of controlled mechanical ventilation

On day 21 of invasive MV, a percutaneous tracheostomy was performed without complications. During this period, the oxygenation and RS mechanics response to PP remained satisfactory. For that reason, we implemented a 7-day intermittent PP strategy. On day 23 of invasive MV, after clinical and radiographic improvement, a new partial support trial was carried out.

### Third period of partial ventilatory support

The patient was only capable of breathing in PSV for 24 hours, given that his clinical state deterioration was rapidly progressive (Figures 2 and 4), which is why we had to reassume controlled MV.

### Fourth period of controlled mechanical ventilation

Although ultraprotective ventilation was used, it was impossible to keep RS mechanics within safe ranges (Figure 4, Figure 1S - Supplementary material).

On day 29 of invasive MV, the patient died due to refractory hypoxemia.

## DISCUSSION

In the present study, we show that classic, basic and advanced monitoring variables generally believed to determine the aptitude to initiate the partial ventilatory support phase were not sufficient to guarantee SB safety. The rapid deterioration of oxygenation and RS mechanics along with the clear radiographic correlation during the SB periods show that P-SILI is feasible even though adequate clinical and ventilator monitoring parameters are present at the beginning of SB.

The C_rs_ was recently proposed by Vaporidi et al. as a bedside parameter to assess the risk of developing high values of ∆P_aw_ during assisted ventilation.^(6)^ Values lower than 20mL/cmH_2_O are unfailingly associated with periods of ∆P_aw_ > 15cmH_2_O; on the other hand, high ∆P_aw_ values are unlikely when C_rs_ is higher than 30mL/cmH_2_O. Thus, below this C_rs_ threshold, it is recommended to use advanced monitoring tools to avoid injurious spontaneous efforts.^(7)^ It is important to consider that, in the aforementioned study, the patients were ventilated with proportional assisted ventilation plus (PAV+), a ventilatory mode whose operative functions and patient-ventilator interactions differ considerably from PSV. However, Bellani et al. recently reported that lower C_rs_ and incremental ∆P_aw_ values during PSV are associated with higher mortality.^(12)^ In line with these recommendations, in our case, the transition phase from controlled to partially assisted ventilation was always initiated with C_rs_ ≥ 30mL/cmH_2_O and ∆P_aw_ lower than 15cmH_2_O. On the other hand, VT was kept within acceptable ranges, even lower than those reported in ARDS patients and well recommended to allow SB.^(13)^ Our findings are coincident with those reported by van Haren et al. and Vaporidi et al. that VT monitoring does not guarantee low ∆P_aw_ and does not allow to discriminate between patients able and unable to breathe spontaneously without potential risks.^(5, 7)^

Previous studies have suggested that the outcomes related to SB during partially supported ventilation depend on oxygenation impairment severity, with clear benefits of SB in mild-moderate ARDS and deleterious effects in severe ARDS.^(2, 14)^ In our case, the patient began every SB period in PSV with a PaO_2_/FiO_2_ greater than 250mmHg.

Three main mechanisms have been presumed to precipitate P-SILI during SB: patient-ventilator asynchrony, overdistension and increased lung perfusion.^(15)^ First, asynchronies were clearly noticed only during one single day during SB periods, so we believe it is unlikely to be the key mechanism of lung injury in this case. Second, even though we have been able to measure RS mechanics using esophageal manometry, the regional increase in transpulmonary pressure (P_L_), especially in dependent lung regions, is highly improbable to be detected when using such global monitoring measures.^(4, 14, 15)^ A clear example of this situation is the occult pendelluft, which describes the gas redistribution movement from nondependent to dependent lung regions, causing local overdistention and tidal recruitment of collapsed tissue, even at protective VT monitoring.^(3, 15)^ However, in our case, this hypothesis could not be confirmed because our monitoring tools were insensitive to the pendelluft phenomenon. Third, regarding increased lung perfusion, an echocardiogram was performed during the ICU stay and showed no alterations; in addition, the patient never presented any clinical signs of fluid overload during the SB periods (i.e., no edema or jugular ingurgitation and no need for antihypertensive medication). Despite that situation, we think that just a modest increase in venous return associated with negative intrathoracic swings might be a reasonable explanation for edema formation, showing rapid and clear changes in X-ray patterns and RS mechanics deterioration during SB, even in the absence of clinical signs of fluid overload and normal cardiac function.^(15)^ Unfortunately, we had no equipment available to measure extrapulmonary water, and a Swan Ganz catheter was not used because the patient was never hemodynamically unstable.

A possible confounding factor related to lung mechanics disorders and oxygenation impairment persistence might be the presence of untreated infections acquired during the ICU stay. However, since the patient´s admission, empirical treatment with cefepime-azithromycin, oseltamivir and trimethoprim-sulfamethoxazole (TMS) in *Pneumocystis carinii* doses plus corticosteroids was started. Moreover, after obtaining positive results from the tracheal aspirates for cytomegalovirus (with a negative viral load in the blood), ganciclovir was initiated. Once bronchoalveolar lavage was performed, he received 4-drug treatment with isoniazid, rifampicin, pyrazinamide and ethambutol, empirically treating tuberculosis, amphotericin covering a possible mycosis and colistin directed against *A. baumannii*. Finally, treatment with the 4 drugs was suspended after obtaining the results of the direct culture and the negative polymerase chain reaction for tuberculosis, and antiretroviral treatment with lamivudine and lopinavir/ritonavir syrup was immediately started.

The retrospective analysis of our case showed that single conventional and advanced monitoring variables at the beginning of the partial ventilatory support phase were not useful to predict the risk and development of P-SILI. The negative outcomes of our report do not intend to discourage the use of respiratory monitoring during SB. In fact, during the SB cycles, we identified a tendency toward deterioration of the RS mechanics, oxygenation and radiographic pattern. The predictive capacity of these tendencies might provide valuable information for the decision-making process; however, this premise should be confirmed through future investigations.

## CONCLUSION

Conventional basic and advanced monitoring variables in this case were not sufficient to identify the aptitude to breathe spontaneously or to predict the risk and development of patient self-inflicted lung injury during partial ventilatory support.
