# Supplementary material for: Lesão pulmonar autoinflingida: é possível identificar o risco? Relato de caso
Source: Rev Bras Ter Intensiva. 2021 Jul-Sep;33(3):461–8. doi: 10.5935/0103-507X.20210061 (PMC8555405; doi:10.5935/0103-507X.20210061)
Supplement: Supplementary file 1 [file rbti-33-03-0461-suppl01.pdf]

Self-inflicted lung injury: is it possible to identify the risk? A case report

Lesão pulmonar autoinflingida: é possível identificar o risco? Relato de caso

Joaquín Pérez[1,2], Javier Hernán Dorado[1], Emiliano Navarro[2,3], Matías Accoce[1,4,5]

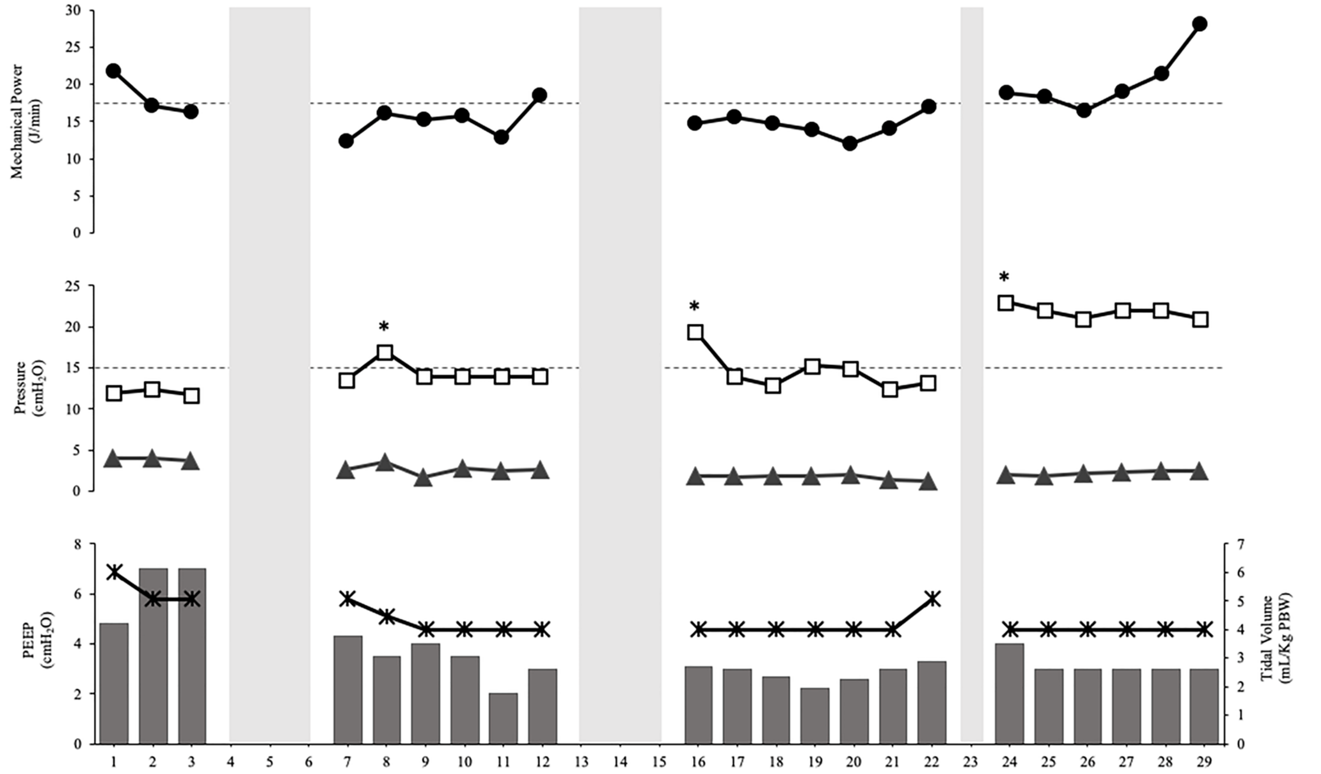

Figure 1S - Evolution of monitoring variables during controlled mechanical ventilation in volume controlled - continuous mandatory ventilation.

Shadow gray areas indicate days of spontaneous breathing under pressure support ventilation, so no data are presented here. Mechanical power (circles) [upper panel]; esophageal driving pressure (triangles) and airway driving pressure (squares) [middle panel]; tidal volume (stars) and positive end expiratory pressure (bars) [lower panel]. Dotted lines indicate threshold values of mechanical power (17J/minute) and airway driving pressure (15cmH₂O).(1-3)

* Of note, airway driving pressure exceeds 15cmH₂O only after spontaneous breathing cycles in pressure support ventilation.

PEEP - positive end expiratory pressure.

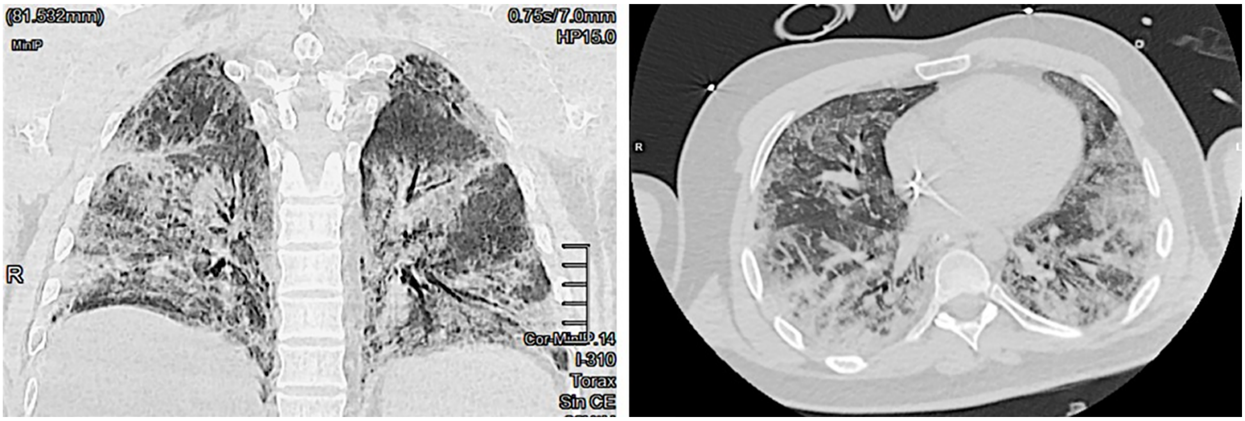

Figure 2S - Chest computed tomography after the first spontaneous breathing cycle under pressure support ventilation.

The progression of lung injury and the appearance of new alveolar and interstitial bilateral infiltrates preferentially in dependent lung regions were clearly noticed.

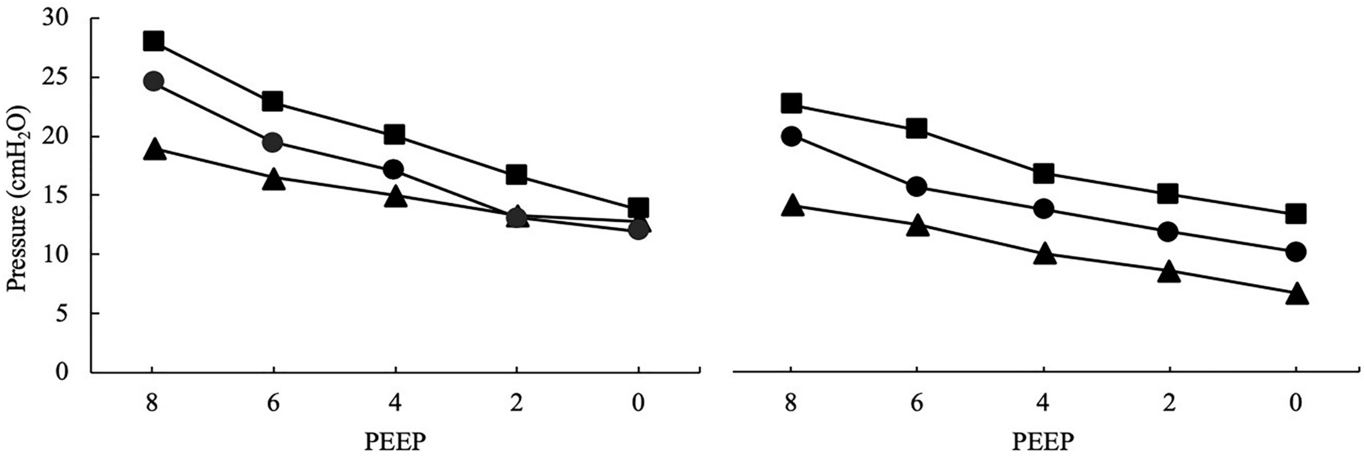

Figure 3S - End inspiratory transpulmonary pressure measured with the classic method (triangles), elastance derived method (circles) and end inspiratory plateau airway pressure (squares) during positive end expiratory pressure titration in volume controlled - continuous mandatory ventilation in supine (left panel) and prone positioning (right panel).
PEEP - positive end expiratory pressure.

REFERENCES

1. Serpa Neto A, Deliberato RO, Johnson AE, Bos LD, Amorim P, Pereira SM, Cazati DC, Cordioli RL, Correa TD, Pollard TJ, Schettino GPP, Timenetsky KT, Celi LA, Pelosi P, Gama de Abreu M, Schultz MJ; PROVE Network Investigators. Mechanical power of ventilation is associated with mortality in critically ill patients: an analysis of patients in two observational cohorts. Intensive Care Med. 2018;44(11):1914-22.
2. Amato MB, Meade MO, Slutsky AS, Brochard L, Costa EL, Schoenfeld DA, et al. Driving pressure and survival in the acute respiratory distress syndrome. N Engl J Med. 2015;372(8):747-55.
3. Vaporidi K, Psarologakis C, Proklou A, Pediaditis E, Akoumianaki E, Koutsiana E, et al. Driving pressure during proportional assist ventilation: an observational study. Ann Intensive Care. 2019;9(1):1.
